# Effects of Messages Emphasizing Environmental Determinants of Obesity on Intentions to Engage in Diet and Exercise Behaviors

**DOI:** 10.5888/pcd10.130163

**Published:** 2013-12-12

**Authors:** Jeff Niederdeppe, Sungjong Roh, Michael A. Shapiro, Hye Kyung Kim

**Affiliations:** Author Affiliations: Sungjong Roh, Michael A. Shapiro, Hye Kyung Kim, Department of Communication, Cornell University, Ithaca, New York.

## Abstract

**Introduction:**

Reducing rates of obesity will require interventions that influence both individual decisions and environmental factors through changes in public policy. Previous work indicates that messages emphasizing environmental determinants increases support for public policies, but some suspect this strategy may undermine motivation to engage in diet and exercise.

**Methods:**

Study 1 involved 485 adults recruited from a shopping mall in New York. Study 2 involved 718 adult members of a Web-based national panel of US adults. Respondents in both studies were randomly assigned to read a story that emphasized environmental determinants of health or a control condition. The stories varied in the extent to which they described the story character as taking personal responsibility for weight management. Logistic regression and ordered logit models were used to test for differences in intentions to engage in diet and exercise behaviors based on which story the participant read. Analyses were also performed separately by participants’ weight status.

**Results:**

In both studies, messages that acknowledged personal responsibility while emphasizing environmental causes of obesity increased intentions to engage in healthy behavior for at least 1 weight status group.

**Conclusion:**

Emphasizing factors outside of personal control appears to enhance rather than undermine motivations to engage in healthy diet and exercise behavior.

## Introduction

Obesity, the third-leading cause of death in the United States (1), is caused by a combination of genetic or biological predispositions, individual decisions about diet and exercise, and societal factors like physical, economic, and social environments (heretofore summarized as “environmental causes”) (2). Reducing rates of obesity in the United States will thus require changes in individual diet and exercise behavior as well as local, state, and federal policy to promote healthy eating environments and increase opportunities for active living (3,4).

Public health organizations have implemented various public communication campaigns to reduce rates of obesity. Traditionally, these efforts have targeted individual behavior change (2). For example, the Centers for Disease Control and Prevention’s VERB campaign, funded at $339 million over 4 years, sought to increase physical activity among youth aged 9 to 13 years by targeting children and their parents with media messages (5). Youth exposed to the campaign were more likely to report engaging in physical activity than those who were not exposed, but the authors did not report effects on rates of childhood obesity (6). The National Cancer Institute’s 5 A Day Program for Better Health aimed to increase consumption of fruits and vegetables through targeted media campaigns and local interventions in schools and worksites (7). The campaign failed to produce population changes in fruit and vegetable consumption, perhaps due to low levels of campaign exposure (8).

Recent national media efforts have shifted attention toward environmental causes of obesity and the necessity for multisector, systems-level changes to address the problem. For instance, an Institute of Medicine (IOM) report emphasizing these factors (2) was accompanied by a 4-part HBO documentary, “The Weight of the Nation.” The documentary mirrored the IOM report’s focus on environmental causes of obesity and emphasized the importance of shared responsibility (both individual and environmental) to reduce obesity rates. Although the effects of this effort are unknown, several studies have found that framing the issue of obesity in terms of its environmental causes can increase public support for obesity-related policies (9,10).

Opponents of policies targeting environmental causes of obesity argue that governmental intervention to reduce obesity reflects a “nanny state” that undermines personal responsibility (11–13). Central to this argument is the premise that policies targeting environmental causes of obesity, as well as strategic communication efforts to promote those policies, will not promote individual decisions about diet and exercise because they absolve people from taking responsibility for their actions (14,15). Another version of this argument suggests that emphasizing environmental factors undermines personal responsibility and reduces intentions to engage in healthy diet and exercise behaviors (16). These perspectives tend to consider personal responsibility (and accompanying emphasis on individual diet and exercise behaviors) and environmental causes (and accompanying emphasis on policy change) as incompatible, requiring messengers to choose one perspective or the other. They also suggest that emphasizing obesity’s environmental causes may at best fail to influence motivation to engage in diet and exercise or, at worst, reduce that motivation.

Studies that have examined these claims, however, suggest that emphasizing nonindividual determinants of obesity can have favorable effects on motivation to engage in healthy behavior (17,18). One study randomly assigned participants to receive, over a 3-month period, 4 print messages that emphasized either individual or social responsibility (partnerships with family, friends, community members, and health professionals) for maintaining a healthy diet. Both messages were successful at increasing fruit and vegetable consumption, although some evidence suggested that the effects of the societal responsibility messages persisted longer (17). Another study randomly assigned participants to read news stories framed to emphasize either individual or environmental causes of obesity. Respondents exposed to the environmentally framed story were more likely than those exposed to the individually framed story to support obesity-related public policies and to intend to engage in obesity-reducing behaviors (18).

These studies provide a useful starting point for understanding how people respond to messages emphasizing obesity’s environmental causes, but they are limited with their reliance on a sample already motivated to engage in health-related behavior (callers to a cancer information hotline) (17) or one largely composed of college students (who have low rates of overweight and obesity) (18). Neither study compared responses between overweight or obese and normal-weight respondents, a key question because overweight and obese adults are at elevated risk of severe health and social consequences (1,2) and thus represent a key target population for obesity-related messages. Furthermore, both studies compared messages that were framed exclusively as individual or social/environmental, but the science of obesity suggests that both individual decisions and environmental factors play roles (3,4). Little is known about effects on intentions or behavior in response to messages highlighting environmental causes of obesity and acknowledging individual responsibility, an approach suggested by some advocates (14,19).

We address these limitations using 2 randomized experiments to test effects of messages emphasizing obesity’s environmental causes, while manipulating various levels of acknowledging individual responsibility, on intentions to engage in diet and exercise behavior (used interchangeably with “behavioral intentions” throughout the article). We hypothesized that messages that strongly acknowledge individual responsibility while maintaining an emphasis on environmental causes would be most likely to influence behavioral intentions (relative to a control group). In the absence of previous studies, we also explored whether these effects were found among overweight/obese respondents, normal-weight respondents, or both.

## Methods

### Participants

Study 1 consisted of 485 adults recruited with signage in a public area of a shopping mall near a mid-sized town in New York State in exchange for a $10 mall gift card. Data were collected on laptop computers during May and June 2010. We did not calculate a response rate for Study 1 because we could not ascertain how many were aware of (and thus eligible for) the study.

Study 2 involved adult members of a Web-based national panel of US adults maintained by GfK Knowledge Networks (GfK) and recruited via random-digit–dialing. Data were collected during July and August 2011. GfK’s panel recruitment rate at the time of our study was 21%. A random sample of 1,462 GfK panelists was invited to participate, of which 718 participants provided informed consent and completed the study. The overall response rate (718/1,462 = 49% cooperation rate by the GfK panel’s 21% panel recruitment rate) was 10%, typical of GfK studies (20). There were no differences by randomized condition in sociodemographics in both studies (*P* values > .19; Table 1). Both studies were approved by Cornell University’s institutional review board.

**Table 1:** Demographics of Participants in Studies of Effects of Messages Emphasizing Environmental Determinants of Obesity on Intentions to Engage in Diet and Exercise Behaviors

Characteristic	Study 1 (n = 485)^a^	Study 2 (n = 718)^a^
**Randomized condition**		
No-exposure control group	0.25 (122)	0.10 (75)
High personal responsibility, emphasizes environmental factors	0.25 (121)	0.43 (310)
Moderate personal responsibility, emphasizes environmental factors	0.25 (123)	0
No personal responsibility, emphasizes environmental factors	0.25 (119)	0.46 (333)
**Age (range, 18–83 y), mean (SD)**	36.4 (16.3)	48.3 (17.0)
**Female sex**	0.57 (275)	0.53 (380)
**Race/ethnicity (respondents could select more than 1 category)**		
White, non-Hispanic or Latino	0.73 (355)	0.74 (533)
Black, non-Hispanic or Latino	0.05 (25)	0.09 (64)
Other, non-Hispanic or Latino	0.15 (74)	0.08 (55)
Hispanic or Latino	0.06 (31)	0.09 (66)
**Highest level of education completed**		
Some high school or less	0.06 (28)	0.08 (56)
High school diploma or equivalent	0.20 (96)	0.29 (206)
Some college or technical school	0.37 (181)	0.30 (217)
College degree or higher	0.37 (180)	0.33 (239)
**BMI (self-reported weight and height, kg/m^2^)**		
Underweight (BMI <18.5)	0.04 (18)	0.01 (10)
Normal weight (BMI ≥18.5 and <25)	0.41 (199)	0.38 (272)
Overweight (BMI ≥25 and <30)	0.29 (138)	0.29 (211)
Obese (BMI ≥30)	0.26 (124)	0.29 (210)
Did not report	0.01 (6)	0.02 (15)
**Political ideology (1 = extremely conservative, 7 = extremely liberal), mean (SD)**	4.1 (1.4)	4.2 (1.5)
**Political party**		
Republican	0.22 (106)	0.28 (198)
Democrat	0.30 (145)	0.37 (264)
Independent	0.29 (141)	0.29 (205)
Something else	0.19 (92)	0.07 (50)
Did not report	0 (1)	0 (1)

Abbreviation: SD, standard deviation; BMI, body mass index.

a Data are expressed as proportion (number) unless otherwise indicated.

### Stimulus materials

**Study 1**

Participants were randomly assigned to view 1 of 4 vignettes. Three of the messages were stories about a middle-aged woman named Michele Wolfe who had managed to lose 11 pounds despite challenges related to weight management, including 1) high cost and lack of access to healthy foods, 2) widespread availability of unhealthy foods, 3) time constraints from a low-income job, and 4) a lack of safe and affordable places for exercise. The second half of the story described efforts by the neighborhood development association to add a local supermarket, bicycle trails, and walking paths that provided residents with easier access to healthful food and opportunities for physical activity. The stories were based on people and community programs described on the website of the Robert Wood Johnson Foundation’s (2008) Commission to Build a Healthier America (Appendices A and B) (21).

The stories explicitly varied the extent to which Michele took personal responsibility for her weight loss (high, moderate, and none). Content related to obesity’s environmental causes and solutions was held constant. The fourth story, a control condition, was unrelated to obesity.

**Study 2**

Participants were randomly assigned to 1 of 9 conditions (8 experimental groups and a no-exposure control group). Those assigned to experimental conditions read near-identical versions of Michele’s story that were used in Study 1, with 2 exceptions. We used a 2 (high vs no personal responsibility) by 2 (Republican or Democratic partisan cue) by 2 (asked to read the story with empathy or rationally) factorial design (Appendices A and B). The empathy directions and partisan cues were intended as exploratory and were thus much shorter and subtler than the personal responsibility manipulation. They were also designed to influence support for obesity-related policies, not behavioral intentions (9,10).

### Measures

After reading the message, respondents in both studies reported their message-related thoughts, character perceptions, emotional responses, beliefs about obesity’s causes and solutions, support for obesity-related policies, behavioral intentions, and demographics. Findings related to effects on thoughts, emotions, causal attributions, and policy support are summarized elsewhere (9).

To gauge behavioral intentions, both surveys asked participants (using a scale ranging from “very unlikely, 1” to “very likely, 5”): “How likely is it that you will . . . [have 5 or more servings of fruits and vegetables; exercise at least 3 times in most weeks; control your diet to lose weight] in the next year?” (22) These items were used as an indicator of motivation to take personal responsibility for weight management, because measures of behavioral intentions strongly predict future behavior (23). We dichotomized each variable, distinguishing between respondents who were somewhat or very likely to engage in the behavior in the next year and those who were not. Most respondents intended to engage in each behavior in both studies, but each behavior was less common in Study 2 (Study 1: fruits and vegetables, 65.2%; exercise, 70.3%; dieting 65.8%. Study 2: fruits and vegetables, 54.2%; exercise, 62.2%; dieting, 57.4%.). We also created a 3-item index counting the number of behaviors a respondent intended to perform in the next year (Study 1: mean, 2.01; standard deviation [SD], 0.99. Study 2: mean, 1.74; SD, 1.07.). We conceptualized this index using a causal indicator measurement model, not an effect indicator model, based on the theoretical prediction that each item would predict subsequent weight loss behavior (23,24).

Respondents reported their weight and height, from which we calculated their body mass index (BMI). We stratified the sample by 2 weight status groups: normal weight (BMI ≥18.5 kg/m^2^ and <25 kg/m^2^) and overweight/obese (BMI ≥25 kg/m^2^).

### Data analysis

We used logistic regression (for each individual behavioral intention item) and ordered logit models (for the index) to test whether randomized condition (which story the participant read) influenced behavioral intentions relative to the control condition. Because we explicitly hypothesized that the high personal responsibility condition would be most likely to influence behavioral intentions, we interpreted a significant coefficient for that condition (relative to control) as support for our overall hypothesis. We also report omnibus tests for all 3 indicators to test whether any other study condition influenced behavioral intentions. We conducted separate models stratified by weight category (normal vs overweight/obese) to examine whether condition effects differed by respondents’ weight status.

## Results

### Manipulation check

We conducted a pilot study (n = 113) with college students before Study 1 to ensure that the manipulations were perceived as intended. Detailed results are available elsewhere, but indicated that participants perceived more emphasis on individual responsibility in the high versus moderate and the moderate versus no personal responsibility conditions, and equivalent emphasis on societal responsibility across messages (9). We also embedded a manipulation check within Study 2 by asking respondents about perceived individual and societal responsibility after reading the story. Results were similar to those observed before Study 1. The individual responsibility manipulation was thus deemed successful.

These empathy and partisan cue manipulations in Study 2 did not induce their intended cognitive outcomes (eg, increasing feelings of empathy; increasing perceived similarity with the character when her political party aligned with the respondent’s), likely a result of the subtlety of their induction. We thus focused on effects of the personal responsibility manipulation.

### Study 1

The high personal responsibility condition produced greater intentions to eat fruits and vegetables and to diet to lose weight, relative to the control condition, among the overall sample (Table 2). Randomized condition did not affect the 3-item index for the overall sample. Similarly, randomized condition did not predict any of the behavioral intention measures among normal-weight respondents. Among overweight/obese respondents, the high personal responsibility condition produced greater intentions to eat fruits and vegetables, greater intentions to engage in regular exercise, and higher scores on the 3-item index than the control group. The moderate personal responsibility message also produced higher scores on the index among overweight/obese participants.

**Table 2:** Differences in Intentions to Engage in Diet and Exercise Behaviors by Condition, Overall and by BMI, in Study 1 of Effects of Messages Emphasizing Environmental Determinants of Obesity on Intentions to Engage in Diet and Exercise Behaviors

Weight Category	Eating Fruits and Vegetables, Logistic OR (*P*)	Regular Exercise, Logistic OR (*P*)	Dieting to Reduce Weight, Logistic OR (*P*)	3-Item Index (0-3), Cumulative Logit Coefficient (*P*)
**Overall sample**				
Randomized condition, *P* (omnibus test; *df* = 3)	.14	.26	.08	.12
No-exposure control group	1 [Reference]			0 [Reference]
High personal responsibility	1.80 (.03)^a^	1.15 (.61)	1.72 (.048)^a^	0.41 (.08)
Moderate personal responsibility	1.21 (.48)	1.72 (.06)	1.62 (.07)	0.44 (.06)
No personal responsibility	1.03 (.91)	1.08 (.78)	1.03 (.92)	0.07 (.77)
Sample n	485	485	485	485
**Normal-weight respondents**				
Randomized condition, *P* (omnibus test; *df* = 3)	.99	.22	.21	.83
No-exposure control group	1 [Reference]			0 [Reference]
High personal responsibility	1.00 (>.99)	0.42 (.07)	1.97 (.13)	−0.19 (.62)
Moderate personal responsibility	0.76 (.88)	0.87 (.77)	1.11 (.79)	−0.15 (.69)
No personal responsibility	1.00 (>.99)	0.61 (.31)	0.79 (.58)	−0.36 (.35)
Sample n	199	199	199	199
**Overweight/obese respondents**				
Randomized condition, *P* (omnibus test; *df* = 3)	.05	.09	.08	.03^b^
No-exposure control group	1 [Reference]			0 [Reference]
High personal responsibility	2.50 (.02)^a^	2.12 (.04)^a^	1.97 (.08)	0.83 (.01)^a^
Moderate personal responsibility	1.26 (.52)	2.41 (.02)^a^	2.58 (.02)^a^	0.76 (.02)^a^
No personal responsibility	0.95 (.88)	1.57 (.20)	1.24 (.56)	0.31 (.32)
Sample n	262	262	262	262

Abbreviation: BMI, body mass index, OR, odds ratio.

a Significant difference from the control group, *P* < .05.

b Significant omnibus test for randomized condition, *P* < .05.

To gauge the magnitude of these effects, we used Stata’s “predict” postestimation command (after the ordered logit models) to estimate the probability of a respondent intending to engage in all 3 behaviors as a function of which vignette that they read. The probability of an overweight/obese respondent engaging in all 3 behaviors was 52% for those who read the high personal responsibility vignette and 51% for those who read the moderate personal responsibility vignette, compared with only 32% of overweight/obese respondents in the control group.

### Study 2

The high personal responsibility condition produced greater intentions to diet to lose weight and higher scores on the 3-item index, relative to the control condition, among the overall sample (Table 3). On the basis of results from postestimation predictions in Stata, the probability of a respondent engaging in all 3 behaviors was 34% for those who read the high personal responsibility vignette compared with 25% of respondents in the control group.

**Table 3:** Differences in Intentions to Engage in Diet and Exercise Behaviors by Condition, Overall and by BMI, in Study 2 of Effects of Messages Emphasizing Environmental Determinants of Obesity on Intentions to Engage in Diet and Exercise Behaviors

Weight Category	Eating Fruits and Vegetables, Logistic OR (*P*)	Regular Exercise, Logistic OR (*P*)	Dieting to Reduce Weight, Logistic OR (*P*)	3-Item Index (0-3), Cumulative Logit Coefficient (*P*)
**Overall sample**				
Randomized condition, *P* (omnibus test; *df *= 2)	.09	0.12	.04^a^	.03^a^
No-exposure control group	1 [Reference]			0 [Reference]
High personal responsibility	0.96 (.89)	1.47 (.15)	1.96 (.009)^b^	0.46 (.049)^c^
No personal responsibility	0.70 (.17)	1.09 (.75)	1.74 (.03)^c^	0.14 (.55)
Sample n	714	716	712	710
**Normal-weight respondents**				
Randomized condition, *P* (omnibus test; *df* = 2)	.52	.05	.03^a^	.02^a^
No-exposure control group	1 [Reference]			0 [Reference]
High personal responsibility	1.02 (.97)	1.37 (.52)	3.75 (.01)^c^	0.77 (.06)
No personal responsibility	0.77 (.57)	0.70 (.47)	2.76 (.06)	0.20 (.61)
Sample n	272	272	271	271
**Overweight/obese respondents**				
Randomized condition, *P* (omnibus test; *df *= 2)	.26	.66	.21	.46
No-exposure control group	1 [Reference]			0 [Reference]
High personal responsibility	1.02 (.96)	1.34 (.37)	1.71 (.10)	0.34 (.25)
No personal responsibility	0.73 (.34)	1.28 (.44)	1.78 (.08)	0.19 (.52)
Sample n	419	421	418	416

Abbreviation: BMI, body mass index.

a Significant omnibus test for randomized condition, *P* < .05.

b *P* < .01.

c Significant difference from the control group, *P* < .05.

Effects on dieting were driven by normal-weight respondents — the high personal responsibility condition produced greater intentions to diet among normal-weight respondents but not overweight/obese respondents. Condition effects on the 3-item index were also significant for normal-weight respondents only (omnibus test *P* = .02).

## Discussion

The messages tested here depicted a story character that models healthy behaviors in a challenging economic and physical environment and receives assistance in these efforts through neighborhood development. When the character was portrayed as taking a high level of personal responsibility for her actions, respondents from at least 1 weight group in both studies increased their behavioral intentions. This finding is consistent with previous research on narrative communication and social cognitive theory, which predicts that exposure to messages featuring characters who model healthy behavior amid environmental barriers can increase self-efficacy and behavioral intentions (25,26). Results are also consistent with 2 previous studies that concluded that emphasizing societal determinants of obesity can have favorable effects on individual motivation to engage in healthy behavior (17,18). Furthermore, we found no evidence that a message emphasizing obesity’s environmental causes, even while ignoring the notion of personal responsibility, reduced healthy behavioral intentions. Consequently, we conclude that the overall body of research to date does not support the argument that emphasizing factors outside of personal control would undermine personal responsibility for healthy behavior.

Although both studies observed positive effects on behavioral intentions for at least 1 weight status group, results were not entirely consistent between studies. Study 1 found that effects were driven by overweight/obese respondents, while Study 2 found effects largely among normal-weight respondents. Fruit and vegetable consumption and regular exercise are healthy behaviors regardless of weight status, but dieting to lose weight is only important for those who are overweight or obese and may be detrimental to those at normal weight but borderline underweight (a BMI near 18.5). Although we can only speculate, it seems plausible that these differences could have been driven by the modality of data collection (at a laptop computer in a public location in Study 1; at a location of the respondents’ choosing, likely at home, in Study 2). Study 1 took place in a central and high-traffic area of the mall. We suspect that obese and overweight respondents may have been more self-conscious about a story related to obesity in this context (27), and thus more motivated by messages strongly emphasizing personal responsibility for weight loss. Future research should test the effect of social cues (exposure to messages in public or private) on responses to messages about public health issues like obesity where a person’s weight status is readily observable.

Two of Study 2’s experimental manipulations were unsuccessful, a fact that may have been driven by the use of a very subtle partisan cue and brief instructions about reading the story with or without empathy. Stronger manipulations may be required to achieve meaningful differences in response to messages. The outcome for all analyses was behavioral intentions, but in the absence of a longitudinal follow-up we do not know what proportion of these respondents followed through on these intentions. Future work should consider longitudinal designs with repeated exposure to multiple messages to examine the durability of these effects.

This study builds on a previous analysis of data from the same randomized experiment that found that exposure to the moderate personal responsibility condition was also effective at increasing the belief that societal factors cause obesity and promoting support for various evidence-based policies to reduce obesity (9). Combined, these analyses suggest that public health advocates should deliberate on the degree to which messages should acknowledge personal responsibility while emphasizing environmental determinants of obesity (14–19). If the goal is to maximize support for public policies targeting environmental determinants, message designers should mention but not overemphasize the role of personal responsibility in achieving healthy weight. If the goal is to maximize healthy behavioral intentions while still acknowledging environmental determinants, which might be important in the absence of a specific policy proposal subject to public discussion or legislative debate, messages could increase the strength by which they acknowledge personal responsibility for weight management. Future research should assess the degree to which acknowledgment of personal responsibility influences intentions and behavior among different weight status groups, particularly obese people at elevated risk of major physical and psychosocial health problems.

## Appendices

### Appendix A: Full Text of Vignettes that Strongly Acknowledge Personal Responsibility

(Differences from the story that did not acknowledge personal responsibility are underlined; text used in the empathy and partisan cue manipulations in Study 2 are [*bracketed and italicized*].)

Study 2 Introduction, not used in Study 1: [*While you are reading this message, try to imagine how Michele feels about what has happened and how it has affected her life. Try to feel the full impact of what Michele has been through and how she feels as a result.*] OR [*While you are reading this message, try to take an objective perspective toward what is described. Try not to get caught up in how Michele feels; just remain objective and detached.*]

Michele, 41, was at high risk of developing diabetes and high blood pressure — probably due to a combination of genetics, personal choices, her environment, and finances, she says. In her family, hearty, inexpensive foods were the suppertime staples. One night they would have spaghetti and meatballs, the next night macaroni and cheese. Her grandmother cooked plenty of pork too. And when it came to exercise, getting outdoors was a risky proposition in crime-ridden, traffic-congested neighborhoods with few safe parks and playgrounds.

After struggling with her weight, Michele has dropped 11 pounds by counting calories, controlling portions, and adopting a diet that moves away from carbohydrates and toward fruits and vegetables.

Michele has always believed that it is her own personal responsibility to be healthy, but it hasn’t been easy.

“At first, I didn’t know how to cook a lot of healthy things,” she says. “The healthier stuff was always more expensive and less likely to fill me up,” says Michele. “With two jobs and the time I spend volunteering for the local committee for the [*Democratic* or *Republican*] Party, I don’t have a lot of time. There are so many cheap and delicious food options in my neighborhood that require little to no preparation at home, they were just easier.”

Fortunately, she’s gotten help. The Neighborhood Development Association (NDA) has helped transform Michele’s neighborhood, making her a big believer that one’s environment influences physical and emotional well-being. NDA has brought a new supermarket and farmer’s market into the neighborhood. These neighborhood resources improve the availability of fresh fruits and produce and make it easier for people like Michele to shop for healthy foods. In addition, NDA’s development of jogging-biking trails, public parks, and a new playground has increased residents’ opportunities for safe physical activity.

“I’m getting much more regular exercise than I used to,” she says. “I can push myself to get the quality of exercise I need. Physical activity is also built into my routine.”

Thanks to NDA, Michele now lives in a true community — where neighbors look out for each other and residents place importance on projects such as landscaping and recycling. Here, she feels comfortable getting out of the house and exercising outside — activities Michele sees as tremendously important for improving her health. This has helped Michele to develop healthier lifestyle habits.

“I look for the specials,” she says, “say eight peppers to a bag or the $1-a-bag special that week.”

Along the way, Michele has gotten her friends and family involved too. She and her co-workers now share recipes for all sorts of healthy foods, like spinach, squash, cabbage, and collard greens. Her children participate in the shopping and cooking too, Michele says, expanding their food repertoire while also keeping mom healthy.

“I won’t say it’s been easy — there have been many challenges along the way. Still, it’s my responsibility to keep myself and my family healthy and to not make excuses,” Michele says. “What NDA has done for the neighborhood has been a huge boost.”

With help from NDA, Michele is now on the path to better health.

### Appendix B: Full Text of Vignettes that Did Not Acknowledge Personal Responsibility

(Text used in the empathy and partisan cue manipulations in Study 2 are [*bracketed and italicized*].)

Study 2 Introduction, not used in Study 1: [*While you are reading this message, try to imagine how Michele feels about what has happened and how it has affected her life. Try to feel the full impact of what Michele has been through and how she feels as a result.*] OR [*While you are reading this message, try to take an objective perspective toward what is described. Try not to get caught up in how Michele feels; just remain objective and detached.*]

Michele, 41, was at high risk of developing diabetes and high blood pressure — probably due to a combination of genetics, her environment, and finances, she says. In her family, hearty, inexpensive foods are the suppertime staples. One night they have spaghetti and meatballs, the next night macaroni and cheese. Her grandmother cooks plenty of pork too. And not long ago, getting outdoors was also a risky proposition in her once crime-ridden, traffic-congested neighborhood with few safe parks and playgrounds.

Recently, however, Michele has dropped 11 pounds, and she didn’t even realize it.

“I haven’t been trying to lose weight, I guess it just happened. I haven’t changed my diet, gone to the gym, or tried to change my habits in any way. With two jobs and the time I spend volunteering for the local committee for the [*Democratic* or *Republican*] Party, there’s no time to count calories or prepare special healthy meals. To tell you the truth, I’m really not concerned with my weight, but I guess I’m glad I lost it.”

Many people like Michele don’t have the time or energy to adopt major lifestyle changes. Instead, community organizations are doing what they can to help improve the health of people who, like Michele, don’t place healthy diet and regular exercise at a very high priority.

One group, the Neighborhood Development Association (NDA), has helped to transform Michele’s neighborhood. She thinks these changes are responsible for her weight loss. NDA’s development of jogging-biking trails, public parks, and a new playground has increased residents’ opportunities for safe physical activity. They have also changed people’s daily routines.

“The neighborhood definitely looks nicer since NDA’s renovation. However, it has changed my commute. Because of all the green spaces there are less parking spots, I now walk about eight blocks on my way to work and on my way home.”

Thanks to NDA, Michele now lives in a true community. Here, she feels more comfortable getting out of the house, even if she’s not intending to exercise. This has helped Michele to improve her health, even though this is not one of her priorities.

“Juggling work, family, and finances, I don’t have the time or the energy to plan my meals ahead of time or squeeze in a workout. I’m worried about providing for my family, so I can’t be jogging around town.”

Despite Michele’s busy schedule, NDA has made some changes that have improved her diet as well. NDA has brought a new supermarket and farmer’s market into the neighborhood. These resources improve the availability of fresh fruits and produce and make it easier for Michele and her neighbors to access cheap and healthy foods.

“I’m not about to spend more money for less food, even if it’s healthier, if we are not going to enjoy it. It just makes sense for us to eat things we like and save money at the same time. However, my family does like some types of vegetables, so, because they’re cheaper at the new supermarket and I pass it on my way home, I’m buying them a bit more often.”

“It’s the responsibility of the community to create a healthier neighborhood for the people living here,” Michele says. “I have other things to worry about — like paying the bills and feeding my family.” Even though Michele hasn’t made her own health a priority, NDA is doing their part to positively influence her well-being.
